# Camellia (*Camellia oleifera* Abel.) Seed Oil Regulating of Metabolic Phenotype and Alleviates Dyslipidemia in High Fat-Fed Mice through Serum Branch-Chain Amino Acids

**DOI:** 10.3390/nu14122424

**Published:** 2022-06-10

**Authors:** Jing Gao, Li Ma, Jie Ma, Siting Xia, Saiming Gong, Yulong Yin, Yongzhong Chen

**Affiliations:** 1 Research Institute of Oil Tea Camellia, Hunan Academy of Forestry, Shao Shan South Road, No. 658, Changsha 410004, China; gaojing.he@163.com (J.G.); shzh276841095@163.com (L.M.); 2 National Engineering Research Center for Oil Tea Camellia, Changsha 410004, China; 3 Key Laboratory of Agro-Ecological Processes in Subtropical Region, Institute of Subtropical Agriculture, Chinese Academy of Sciences, Changsha 410125, China; 4 College of Animal Science and Technology, Hunan Co-Innovation Center of Animal Production Safety, Hunan Agricultural University, Changsha 410127, China; jie_ma2022@stu.hunan.edu.cn (J.M.); siting_hsia@stu.hunau.edu.cn (S.X.); 673628317gsm@gmail.com (S.G.)

**Keywords:** camellia (*Camellia oleifera Abel.*) seed oil, dyslipidemia, branched-chain amino acid, metabolic phenotype, PPARγ

## Abstract

Camellia (*Camellia oleifera Abel.*) seed oil (CO) has been shown to effectively reduce the blood lipid level of its host due to its fatty acid content, but the specific molecular mechanism associated with the metabolic phenotype after digestion is not clear. Here, we further investigated the relationship between branched-chain amino acids (BCAA) and the metabolic phenotype that may exhibit the anti-dyslipidemia effect of CO on mice fed a high-fat diet for 30 day C57BL/6J male mice were allocated to three groups: the control group (Cont), the high-fat feed group (HFD), and a high-fat feed group with CO treatment (CO). A serum sample was collected to detect lipid biomarkers and BCAA concentration. Notably, Low-density lipoprotein (LDL), Total Cholesterol (TC), and Triglycerides (TG) showed a significant decrease, whereas High-density lipoprotein (HDL) increased in CO mice but not in the HFD group. The concentration of Isoleucine (Ile), leucine (Leu), and valine (Val) was similar between the Cont and CO groups compared with the HFD group, exhibiting an inhibition induced by CO in mice fed with a high-fat diet. A metabolic phenotype from serum examined by non-targeted metabolite analysis using UHPLC/MS showed most metabolites exhibited lipid and BCAA metabolism. The results indicated that CO treatment notably regulated the metabolism of arachidonic acid and steroid biosynthesis in response to HFD-induced dyslipidemia. In addition, the expression of PPARγ genes that correlated with the BCAA and serum lipid biomarkers were compared, and significant inhibition was noticed, which might lead to the potential exposure of the anti-dyslipidemia mechanism of CO in HFD-fed mice. In conclusion, the expression of PPARγ genes, serum lipid level, BCAA concentration, and the metabolic phenotype was significantly positive in correlation with a high-fat diet, whereas oral CO improved the biomarkers and metabolism of some specific serum metabolites in HFD-fed mice.

## 1. Introduction

Dyslipidemia is a disease that can occur at all ages, mainly induced by abnormal lipid metabolism of the host, resulting in accumulation of lipid in the cardiovascular system, causing elevated LDL, TG, and TC, or decreased HDL [1,2]. Furthermore, dyslipidemia is reported to be responsible for other cardiovascular diseases, such as heart failure and type 2 diabetes mellitus [3,4]. Moreover, recent studies found that dyslipidemia is closely associated with obesity [5]. Studies in animal models and human participants have exhibited that diet constituents and habits markedly influence the blood lipid biomarkers of the host [6,7]. A diet that contains mainly unsaturated fatty acids, especially monounsaturated fatty acids, such as oleic acid, rather than saturated fatty acids is shown to promise a lower level of LDL [8]. Moreover, fats from plants are more positively correlated with normal lipid biomarkers than those from animal sources [9]. These findings indicate an interaction between diet fatty acids and dyslipidemia. Furthermore, Li et al. reported the anti-dyslipidemia effect of camellia oil on high-fat diet-induced non-alcoholic fatty liver disease in a rat model [10]. We further investigated the potential effect mechanism of CO in HFD-induced dyslipidemia, which is probably closely linked with the metabolism of the lipid, particularly, the mammalian target of the rapamycin (mTOR) signal pathway and gut microbiota-mediated inhibition of lipid synthesis.

There is growing evidence that BCAAs are involved in lipid metabolism regulation, and increasing attention is being paid to the addition of BCAAs to people’s daily diets [11,12]. Isoleucine (Ile), leucine (Leu), and valine (Val) are recognized as BCAA and have striking regulation in influencing lipid metabolism and gut microbiota [13,14,15]. More recently, several studies have elucidated that the serum BCAA level contributes to the onset of type 2 diabetes mellitus and has a strong effect on insulin resistance by using chromatography MS/MS (LC-MS/MS) analysis [16]. In addition, BCAA and its metabolites were found to increase the incidence of obesity [17]. Moreover, BCAA was implied to modulate lipid accumulation by regulating the activity of the mTOR signal pathway, and several studies suggest that the nuclear receptor peroxisome proliferator-activated receptor γ (PPARγ) modulates BCAA metabolism to interrupt type 2 diabetes [18,19]. Since BCAA has such a close link with lipid metabolism and some cardiovascular diseases, we wonder whether the serum BCAA level and its metabolites were concerned with the anti-dyslipidemia effect of CO in high-fat diet feed mice. 

In the current study, we further conducted the anti-dyslipidemia action of CO by figuring out the regulation of serum BCAA concentration and its metabolites on lipid metabolism. We concluded that CO regulating of the metabolic phenotype alleviates dyslipidemia in high fat-fed mice; in particular, BCAA mediated the regulation of arachidonic acid and steroid biosynthesis metabolism. 

## 2. Materials and Methods

### 2.1. Fatty Acids Profile of CO

CO (Xianglin No.210 used in this experiment is the main promoted strain in Hunan Province) was extracted from CO seeds that were selected from the National Engineering Research Center of Oil-tea Camellia, Hunan Academy of Forestry, by the low-temperature cold press extraction method. The chemical composition of CO was determined by GC-MS analysis (Guangzhou GRG Metrology & Test Co., Ltd. Changsha, China) 

### 2.2. Animal Procedure 

Thirty C57BL/6J male mice (aged 6 weeks, SLAC Laboratory Animal Central, Changsha, China) were divided into three groups. One was given a normal diet (Cont), and the remaining two groups were provided a high-fat diet that lasted for 30 day to establish a dyslipidemia model in mice. After the modeling time, mice that ingested the high-fat diet were weighed and randomly apportioned into two groups: the HFD group (mice fed a high-fat diet and given intragastric administration of normal saline, 200 μL) and the CO group (mice fed a high-fat diet and given intragastric administration of CO, 9 g(kg-day). The authors’ previous results showed that 9 g (kg-day) significantly increased the growth performance of normal C57BL/6J male mice compared to 6 g (kg-day) and 12 g (kg-day) by CO gavage. All animals were housed in the cage of ten mice and promised a free diet and water for 6 weeks to decide the regulation effect of CO on serum lipid in high-fat diet-induced dyslipidemia mice. Our experiment proceeded in compliance with the Chinese guidelines for animal welfare. The experimental protocol was approved by the Animal Care and Use Committee of the Institute of Subtropical Agriculture, Chinese Academy of Sciences, ethical approval code: ISA2017030523. 

### 2.3. Serum Lipid Biomarkers Parameters

A Cobas c-311 colter chemistry analyzer was conducted to measure the lipid biomarkers in serum with the commercially available porcine-specific kits by atomic absorption spectrometry—high-density lipoprotein (HDL) (Gen.3, 200Tests, cobas c, Integra Reagents, kits); low-density lipoprotein (LDL) (Gen.2, 175Tests, cobas c, Integra Reagents, kits); triglycerides (TG, 20767107322); and total cholesterol (TC, 030397731900).

### 2.4. BCAA Concentration Assay 

Serum samples were collected for analysis of Val, Ile, and Leu concentration by High-speed Amino Acid Analyzer L-8900 (Hitachi, Japan).

### 2.5. RT-PCR and Western Blot Analysis

PPARγ genes and β-actin sequences were designed by Primer5.0 software based on the primer design principles (Table 1). Total PPARγ proteins from the liver sample were determined by chosen (ab272718, 1:1000 ABclonal) antibodies to conduct the level of total proteins.

### 2.6. Metabolomics Analyses of Serum 

Serum samples (100 μL) were collected in the Eppendorf Micro Test tubes and resuspended with prechilled 80% methanol, and were then incubated on ice for 5 min and centrifuged at 15,000× *g*, 4 °C for 20 min. The supernatant was diluted to a final concentration containing 53% methanol by LC-MS grade water and injected into the Liquid Chromatography with Mass Spectrometry (LC-MS/MS) system analysis. UHPLC-MS/MS analyses were performed using a Vanquish Ultra High-Performance Liquid Chromatography (UHPLC) system (Thermo Fisher, Germany), coupled with an Orbitrap Q Exactive TM HF mass spectrometer (Thermo Fisher, Germany) in Novogene Co., Ltd. (Beijing, China). Samples were injected onto a Hypesil Gold column (100 × 2.1 mm, 1.9 μm) using a 17-min linear gradient at a flow rate of 0.2 mL/min. The eluents for the positive polarity mode were eluent A (0.1% FA in Water) and eluent B (Methanol). The eluents for the negative polarity mode were eluent A (5 mM ammonium acetate, pH 9.0) and eluent B (Methanol). The raw data files generated by UHPLC-MS/MS were processed using the Compound Discoverer 3.1 (CD3.1, Thermo Fisher) to perform peak alignment, peak picking, and quantitation for each metabolite. Statistical analyses were performed using the statistical software R (R version R-3.4.3), Python (Python 2.7.6 version), and CentOS (CentOS release 6.6). The metabolites were annotated using the KEGG database (https://www.genome.jp/kegg/pathway.html, accessed on 19 August 2021), and the LIPID Maps database (http://www.lipidmaps.org/). We applied univariate analysis (*t*-test) to calculate the statistical significance (*p*-value). The metabolites with VIP > 1 and *p*-value < 0.05 and fold change ≥2 or FC ≤ 0.5 were considered to be differential metabolites, whereas a *p*-value < 0.05 was identified as statistically significant. The functions of these metabolites and metabolic pathways were studied using the KEGG database. Metabolites were identified by variable importance for projection (VIP) values and *p* values. The functions of these metabolites and metabolic pathways were studied using the KEGG database. The metabolic pathways enrichment of differential metabolites was performed. When the ratio was satisfied by x/*n* > y/*N*, the metabolic pathway was considered an enrichment; when the *p*-value of the metabolic pathway < 0.05, the metabolic pathway was considered a statistically significant enrichment.

### 2.7. Statistical Analyses 

Data statistical analyses were operated under the combination of Levene’s test and the student’s *t*-test (IBM SPSS 21.0 software IIME software (Version 1.7.0)) in the one-way analysis of variance (ANOVA). All data are expressed as mean ± SEM, and the *p*-value was considered to be significant when *p* < 0.05.

## 3. Results

### 3.1. Fatty Acids Profile of CO

To determine the fatty acid composition of CO, 49 kinds of free fatty acid substances were measured. We found that CO consists of monounsaturated fatty acids (82.41%), linoleic acid (6.27%), and a small amount of saturated fatty acids (11.33%) (Table 2).

### 3.2. CO Alleviates Dyslipidemia of HFD-Fed Mice

**Figure 1:**
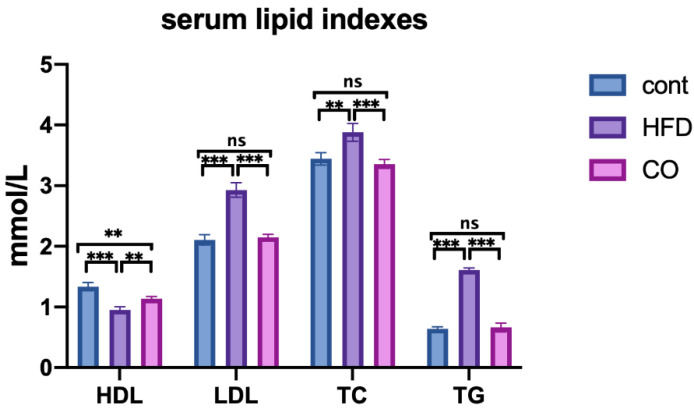
Effect of CO treatment on serum levels of lipid in HFD-fed mice. The concentration of High-density lipoprotein (HDL), Low-density lipoprotein (LDL), Total Cholesterol (TC), and Triglycerides (TG) in mice. Data are expressed as the mean ± SEM (*n* = 10), ** *p* < 0.01, *** *p* < 0.001, ns *p* > 0.05.

To determine whether CO has an anti-dyslipidemia effect, we detected the concentration of HDL, LDL, TC, and TG in the serum of mice. It turned out that CO significantly resumed the serum concentration of LDL, TC, and TG in mice that were fed a high-fat diet (*p* < 0.001), but failed to increase the serum HDL level compared to the HFD group (Figure 1). These results hinted that CO largely alleviated dyslipidemia in HFD-mice, and further experiments should be conducted due to the failure recovery of serum HDL. 

### 3.3. CO Inhibits the Serum Concentration of BCAA in HFD-Fed Mice

**Figure 2:**
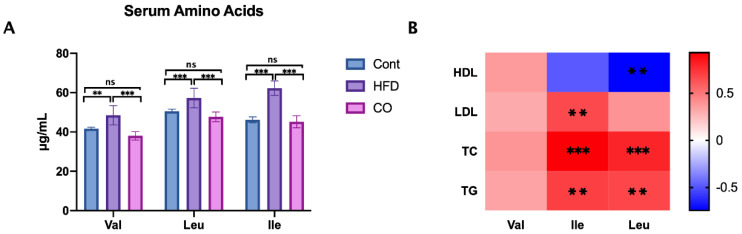
Effect of CO treatment on serum levels of BCAA in HFD-fed mice. (**A**) the concentration of BCAA in serum; data are expressed as the mean ± SEM (*n* = 10), ** *p* < 0.01, *** *p* < 0.001, ns *p* > 0.05; (**B**) the Spearman’s correlation analysis was conducted between serum lipid biomarkers and serum BCAA concentration, and the correlation coefficient was used for the heat map: **, *p* < 0.01; ***, *p* < 0.001.

BCAA has been identified to play a key element in some cardiovascular diseases, which are disturbed in type 2 diabetic mice or obesity models [20]. Thus, we collected serum samples and determined the concentration of valine, leucine, and isoleucine in three groups. In line with our expectations, the serum valine, leucine, and isoleucine levels exhibited a significant reduction in HFD-mice that were treated with CO compared to that of amino acids in the HFD group (*p* < 0.001), which suggested that the anti-dyslipidemia effect of CO may be closely associated with the serum BCAA concentration (Figure 2A). 

To explore whether serum BCAA concentration was involved in the serum lipid biomarkers, we conducted a Spearman correlation analysis between the serum BCAA concentration and serum lipid biomarkers. Interesting, the result was within our expectations. Ile concentration was found positively corrected with serum LDL (*p* < 0.01), TC (*p* < 0.001), and TG (*p* < 0.001) levels; Furthermore, Leu exhibited a positively corrected with serum TC (*p* < 0.001) and TG (*p* < 0.01) levels, and a negatively corrected with HDL (*p* < 0.01) level. Thus, the concentration of the serum BCAA, especially Ile and Leu concentrations, was closely associated with serum lipid biomarkers. The serum lipid biomarkers were disordered within the HFD group, while balanced through the administration of CO (Figure 2B).

### 3.4. CO Affected the Expression of PPARγ in HFD-Fed Mice

**Figure 3:**
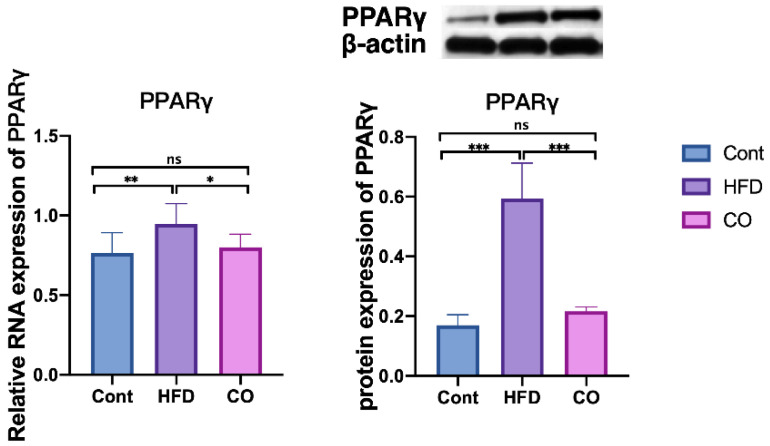
Effect of CO treatment on serum levels of PPARγ in HFD-fed mice. Data are expressed as the mean ± SEM (*n* = 10), * *p* < 0.05, ** *p* < 0.01, *** *p* < 0.001, ns *p* > 0.05.

As we mentioned before, PPARγ act as the core regulator of lipid metabolism, and notably influenced the concentration of BCAA in Type 2 diabetes [18]. To figure out the potential associations between BCAA and the anti-dyslipidemia effect of CO, we further performed the relative expression of the PPARγ gene in the liver of mice. Interestingly, CO treatment on HFD-mice markedly prevented the protein expression of PPARγ (*p* < 0.001), whereas the relative mRNA expression of PPARγ exhibited a similar decrease compared to the HFD mice (Figure 3). Altogether, the serum concentration of lipids and BCAA was detrimental in the HFD-fed mice, which was significantly ameliorated by the administration of CO. 

### 3.5. CO Altered the Metabolic Phenotype of Serum in HFD-Fed Mice

To further determine the underlying anti-dyslipidemia mechanism of CO that is mediated by BCAA, we conducted a non-targeted mass spectrometry-based metabolomics profiling analysis of serum. A total of 1917 metabolites were detected among the three groups. Fortunately, 668 metabolites were fully matched with the KEGG and HMDB database searching, and 324 metabolites were selected as potential biomarkers based on the principle of Variable importance for projection (VIP) values > 1, *p*-value < 0.05. 

**Figure 4:**
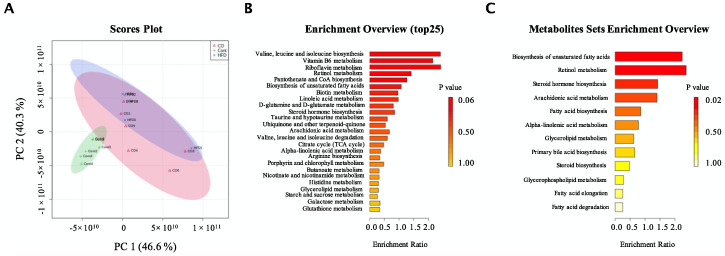
Metabolic phenotype of serum in mice. (**A**) Principal component analysis (PCA) showed differences in metabolites of serum samples in mice between the control, the HFD group, and the CO group. The green area represents the cumulative from the control mice, the pink circle exhibits the cumulative metabolites belonging to the HFD group, and the purple area shows the cumulative metabolites in CO treatment mice; (**B**) Metabolic pathway enrichment analysis. Overview of metabolites that were enriched in mice among three groups; (**C**) Metabolic pathway related to lipid enrichment analysis. Overview of metabolites related to lipid that was enriched in mice among three groups. Each dietary group comprised *n* = 6 mice.

Interestingly, from the intuitive description of differences in metabolic patterns and clustering results between groups, the PCA analysis indicated that the HFD and the CO mice have about 30% overlap, whereas the Cont group was completely separated from the other groups (Figure 4A). The contamination during sampling may have contributed to this 30% overlapping phenomenon between the HFD and the CO mice. To determine the pathway that metabolites were common in, we conducted a KEGG analysis. Our data implied that valine, leucine, and isoleucine biosynthesis, vitamin B6 metabolism, riboflavin metabolism, retinol metabolism, pantothenate, CoA biosynthesis, biosynthesis of unsaturated fatty acids, biotin metabolism, linoleic acid metabolism, steroid hormone biosynthesis, taurine and hypotaurine metabolism, ubiquinone and other terpenoid-quinone, arachidonic acid metabolism, valine, leucine and isoleucine degradation, and citrate acid metabolism significantly reacted to HFD and treatment of CO (Figure 4B). The synthesis of branched-chain fatty acid abundance was affected, which also corresponds to our previous results. Moreover, we then further analyzed metabolites by the KEGG under the categorization of metabolites as lipids considering the anti-dyslipidemia effect of CO was closely associated with lipid metabolism, and we found that CO markedly affected the biosynthesis of unsaturated fatty acids, retinol metabolism, steroid hormone biosynthesis, arachidonic acid metabolism, fatty acid biosynthesis, alpha-linoleic acid metabolism, glycerolipid metabolism, primary bile acid biosynthesis, steroid biosynthesis, glycerophospholipid metabolism, fatty acid elongation, and fatty acid degradation in HFD-mice (Figure 4C). ln summary, mice fed the high-fat diet affected several metabolisms such as BCAA, lipid, fatty acids, and steroids, whereas administration of CO could alter this trend. 

**Figure 5:**
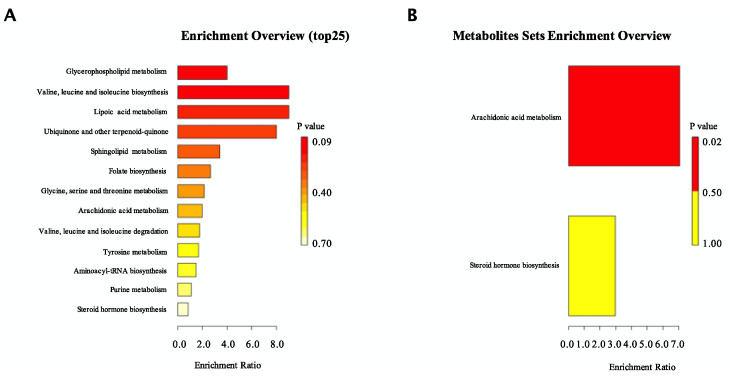
Effect of CO treatment on serum metabolic phenotype in HFD-fed mice. (**A**) Metabolic pathway enrichment analysis. Overview of metabolites that were enriched in mice treated with CO fed a high-fat diet compared to the HFD group; (**B**) Metabolic pathway related to lipid enrichment analysis. Overview of metabolites related to lipid that was enriched in mice treated with CO fed a high-fat diet compared to the HFD group. Each dietary group comprised *n* = 6 mice.

To better explore the underlining molecular mechanism of the anti-hyperlipidemic effect of CO, we performed a comparison of the metabolites between the HFD mice and the CO group. We obtained a total of 227 metabolites; 27 metabolites identified as putative important biomarkers, with 7 metabolites exhibiting an upward expression and 19 metabolites down expressed (Supplementary Table S1). Compared to HFD, a 30 d administration of CO prominently enhanced the concentration of Stercobilin, Glycoursodeoxycholic acid, Muricholic acid, Menaquinone, Phosphoethanolamine, Stearoyl ethanolamide, and Glycerophospho-N-palmitoyl ethanolamine, and reduced the concentration of Noradrenaline, Prostaglandin H2, Mesalamine, Sepiapterin, Lipoic acid, lipoamide, Picrotin, Isoferulic acid, Citicoline, isoleucine, 7-Methyladenine, Testosterone, Hydroquinone, Dimethylglycine, Norbuprenorphine, Oxoamide, and Acetophenone. After an enrichment analysis, we found CO mainly regulated glycerophospholipid metabolism, valine, leucine and isoleucine biosynthesis, lipoic acid metabolism, ubiquinone and other terpenoid-quinone biosynthesis, sphingolipid metabolism, folate biosynthesis, glycine, serine and threonine metabolism, arachidonic acid metabolism, valine, leucine and isoleucine degradation, tyrosine metabolism, aminoacyl-tRNA biosynthesis, purine metabolism, and steroid hormone biosynthesis (Figure 5A). Based on the result conducted from the KEGG-lipid database, CO was closely contributed to the arachidonic acid metabolism and steroid hormone biosynthesis (Figure 5B).

### 3.6. BCAA Correlated with Metabolic Phenotype and Serum Lipid Levels

**Figure 6:**
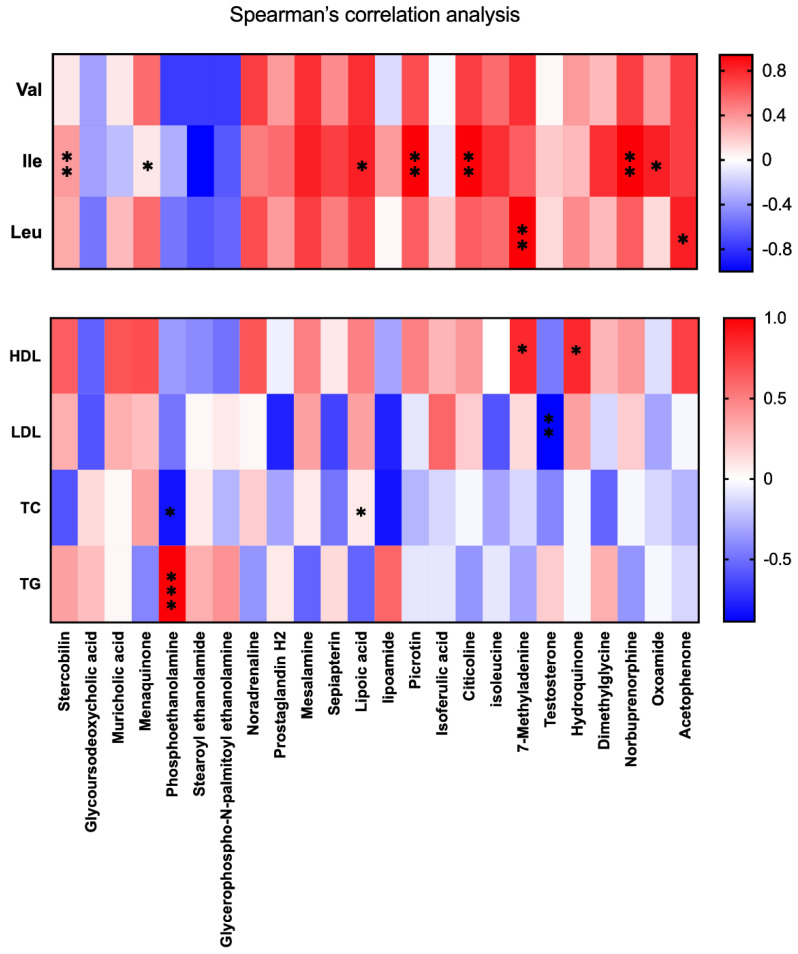
Correlation analysis of metabolic phenotype among serum BCAA concentration and serum lipid biomarkers. Spearman’s correlation analysis was conducted, and the correlation coefficient was used for the heat map: * *p* < 0.05; ** *p* < 0.01; *** *p* < 0.001. Each dietary group comprised *n* = 6 mice.

To further investigate whether the metabolic phenotype alteration (27 putative important biomarkers from CO and HFD group) in HFD mice treated with CO is mediated by BCAA, a Spearman’s test was conducted. Surprisingly, Ile showed a positive correction with the relative abundance of Stearoyl ethanolamide (*p* < 0.01), Mesalamine (*p* < 0.05), Lipoic acid (*p* < 0.05), Picrotin (*p* < 0.01), Citicoline (*p* < 0.01), Norbuprenorphine (*p* < 0.01), and Oxoamide (*p* < 0.05), whereas Leu was positively corrected with the relative abundance of 7-Methyladenine (*p* < 0.01) and Acetophenone (*p* < 0.05). Moreover, we also performed a Spearman’s test between serum lipid biomarkers and the 27 putative important biomarkers from CO and HFD groups, and the results indicated that the relative abundance of 7-Methyladenine and Hydroquinone was positively correlated with serum HDL concentration (*p* < 0.05). Testosterone was negatively correlated with serum LDL concentration (*p* < 0.05) and Phosphoethanolamine exhibited a negative correlation with TC (*p* < 0.05), but positively correlated with TG (*p* < 0.001), whereas lipoamide was positively correlated with TC (*p* < 0.05) (Figure 6). A further experiment should be conducted because no correction was noticed between Val and serum lipid biomarkers or the serum metabolic phenotype. 

## 4. Discussion 

Our previous data indicated that the administration of CO significantly improves HFD-induced hyperlipidemia by reprogramming the gut microbiota composition and inhibiting the mTOR pathway and biosynthesis of lipid-related genes [21]. BCAA is an important nutrient metabolism signaling molecules and was reported to act as an important regulator role in lipid metabolism, obesity, insulin resistance, and metabolic dysfunction in rodents [15,22,23]. Moreover, Ile and leu are superior to the mTOR signal pathway. Although many studies indicated that BCAA concentration is closely linked with several cardiovascular diseases, rarely have experiments been conducted to figure out the relationship between BCAA and hyperlipidemia [24]. CO is a vegetable cooking oil with high nutritional value, and our previous tests have proven that it has a very good anti-hyperlipidemic effect. To explain the potent mechanism of the anti-hyperlipidemic effect of CO in response to HFD, we further investigated that CO may regulate serum BCAA concentration in HFD-fed mice; importantly, the regulation of serum BCAA concentration by CO is closely related to the metabolic phenotype and serum lipid biomarkers. The present study implied that administration of CO markedly influenced the metabolic phenotype and alleviated dyslipidemia in HFD-mice for 6 weeks, which is mediated by the concentration of serum BCAA. 

BCAA was shown to be positively associated with TG and inversely associated with HDL-C in people with type 2 diabetes mellitus disease, which was consistent with our study [25,26]. In this experiment, we found that CO notably reduced the concentration of valine, Leu, and Ile in serum, especially Ile concentration, which exhibited a positive correction with LDL, TC, and TG levels. Leu was improved and had positive correction with TC and TG levels, whereas it was negatively corrected with HDL, but no correction was noticed between Leu and LDL levels. Although lots of studies indicated that Val has been closely linked with amino acid fluxes and lipid metabolism, our results implied that Val did not involve in the regulation of serum lipid biomarkers in HFD-fed mice treated by CO [27].

Interestingly, the activity of the mTOR pathway was closely involved in the regulation of PPARγ in subcutaneous adipose tissue. Our previous study implied that CO improved dyslipidemia and alleviated lipid accumulation by inhibiting the mTOR pathway [19]. In addition, PPARγ was reported to have a modulation effect on BCAA metabolism in type 2 diabetes, and PPARγ ligands have a close association with BCAA metabolism in adipose tissue [18,19,28]. However, it is still unclear whether BCAA concentration influences the expression of PPARγ to further regulate lipid metabolism in a hyperlipidemic mice model. In this study, the expression of liver PPARγ was significantly alleviated in CO-treated HFD mice compared to the HFD mice, which is similar to the previous conclusion. In summary, 6 weeks of administration with CO inhibited the serum BCAA concentration, and further suppressed the expression of the liver PPARγ, thus improving hyperlipidemia in HFD mice. 

The KEGG pathway analysis of serum metabolites in the present study confirmed our suspicions that metabolites in the present study, all chosen potent biomarkers, were enriched in the valine, leucine and isoleucine biosynthesis, vitamin B6 metabolism, riboflavin metabolism, retinol metabolism, pantothenate and CoA biosynthesis, biosynthesis of unsaturated fatty acids, biotin metabolism, linoleic acid metabolism, steroid hormone biosynthesis, taurine and hypotaurine metabolism, ubiquinone and other terpenoid-quinone, arachidonic acid metabolism, valine, leucine and isoleucine degradation, and citrate acid metabolism in all three groups. Both the biosynthesis and degradation of BCAA were regulated by the treatment of CO. Moreover, vitamin B6, riboflavin, retinol, and pantothenate are water-soluble vitamins, which play an important role in the regulation of lipid metabolism and amino acids metabolism. A diet supplemented with BCAA under vitamin B6 deficiency increased the accumulation of TG, and the underlying reason for amino acid mechanism imbalance and serum lipid accumulation may be mediated by vitamin B6 deficiency [29]. Riboflavin and retinoic are widely used in the prevention of hyperglycemia, hypertension, and diabetes mellitus. The disorder of the riboflavin and the retinoic mechanism is closely associated with lipid and glucose mechanisms in the liver and adipose tissue [30,31,32]. Pantothenate and CoA biosynthesis are strongly involved in the metabolism of the fatty acids, and a UPLC-MS-based urine nontargeted metabolic profiling implied that pantothenate and coenzyme A biosynthesis pathway alteration was the most prominent in diabetic kidney disease (a major prevalent chronic microvascular complication of type 2 diabetes) subjects [33]. Linoleic acid is competent in CO and has been widely proved to be linked with liver lipid accumulation [34,35]. Additionally, the current study indicated that linoleic acid metabolism is also involved in serum lipid levels. Steroid hormone biosynthesis, taurine and hypotaurine metabolism, ubiquinone and other terpenoid-quinone, arachidonic acid metabolism, and citrate acid metabolism are key regulators related to amino acids, liver, and serum lipid mechanisms [36,37,38,39,40]. In short, our current results indicated CO altered endogenous metabolites mechanism closely associated with BCAA, vitamins, glucose, lipids, amino acids, and fatty acid mechanisms in response to the HFD-diet in mice. 

In addition, the KEGG enrichment analysis between the CO and HFD groups revealed that CO strongly influenced the BCAA, glucose, lipids, amino acids, and fatty acid mechanisms in HFD mice. Moreover, we found arachidonic acid metabolism and steroid hormone biosynthesis were two distinguished pathways under the interposition by CO when enriching chosen metabolites in the KEGG-lipid database. CO inhibited the steroid hormone biosynthesis and arachidonic acid metabolism in HFD-mice, which was demonstrated by the lower level of Prostaglandin H2 in the serum. In addition, the previous study indicated that Prostaglandin H2 maybe participate in the regulation of coronary arteries, thus improving or normalizing endothelial dysfunction and platelet monocyte adhesion in hyperlipidemia patients [41]. In the CO group, the biosynthesis of the steroid hormone differed from that in the HFD mice, which could be explained by the decreased concentration of Citicoline and Isoferulic acid. These two metabolites modulated the activity of PARP enzymes to further regulate the expression of PPARγ. As mentioned before, PPARγ has an inhibitor role in the incidence of non-alcoholic fatty liver disease, hyperlipidemia, and obesity [42,43]. The reduction of Citicoline and Isoferulic acid in CO mice may contribute to the inhibition of PPARγ expression, and hyperlipidemia induced by a high-fat diet. According to the correction analysis of serum lipid and putative important biomarkers, we figured out that the relative abundance of 7-Methyladenine and Hydroquinone was positively correlated with serum HDL concentration. However, the concentration of 7-Methyladenine and Hydroquinone was down expressed in the CO group compared to the HFD. Furthermore, Spearman’s test showed that 7-Methyladenine also positively correlated with the serum Leu level, which is consistent with reducing the concentration of the serum Leu in the CO group. According to Spearman’s test study, the Leu level did not correct with serum HDL and LDL levels. Moreover, 7-Methyladenine is one indicator of exposure to methylation agents. Further study must be conducted to explain the effect of 7-Methyladenine on serum lipid levels through serum Leu concentration. Testosterone contributes to lipid mechanism disorder and serum lipid levels in male animals, and testosterone was demonstrated to have a negative correlation with serum LDL concentration in our study [44]. The relative abundance of Phosphoethanolamine was reduced by CO in HFD mice in our study. Phosphoethanolamine is an abundant signaling phospholipid and participated in the mechanism of glucose, while exhibiting a negative correlation with TC but was positively corrected with TG [45]. Further experiments are needed to demonstrate this opposite regulatory mechanism. Lipoamide acts on 3T3-L1 adipocytes and significantly increases the number and volume of adipocyte mitochondria, which may have potential therapeutic applications in obesity and diabetes [46]. Furthermore, the treatment of CO notably remitted the abundance of Lipoamide in HFD mice. More importantly, lipoamide was positively correlated with serums Ile and TC, which means the reduction of the serum Ile by CO treatment induced the mitigation of lipoamide, thus alleviating the concentration of TC. 

We conducted Spearman’s test to figure out the regulation of BCAA on the alternation of metabolic phenotype in HFD mice. Surprisingly, the result showed mainly serum Ile and Leu levels influenced the metabolic phenotype. Ile was indicated as positively corrected with the relative abundance of Stearoyl ethanolamide, Mesalamine, Lipoic acid, Picrotin, Citicoline, Norbuprenorphine, and Oxoamide. Moreover, Ile was found to decrease in CO treatment mice compared to HFD mice. Lipoic acid supplementation consistently causes lipid lowering in both blood and tissue pools, and the concentration of Ile and Lipoic acid was reduced in HFD mice with CO treatment [47,48]. Picrotin is an inhibitor of Gamma-aminobutyric acid, whereas Gamma-aminobutyric acid exhibits an efficient anti-dyslipidemia effect [49]. The reduction of Picrotin in our study may contribute to the anti-dyslipidemia effect of CO. However, we did not notice any correction between Val and the metabolic phenotype in our study, which indicates that CO regulating of the metabolic phenotype alleviates dyslipidemia in high fat-fed mice through serum BCAA, especially mediated by serum Ile and Leu levels. 

## 5. Conclusions

**Figure 7:**
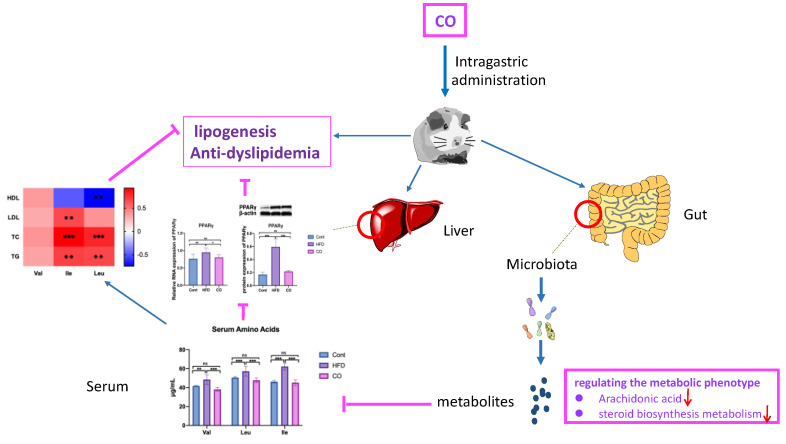
CO enhances the concentration of HDL, but decreases the LDL, TG, and TC levels in serum of HF-fed mice by regulating liver lipid metabolism through the positive correlation between alteration of metabolic phenotype and inhibition of serum BCAA concentration, especially in Ile and Leu. Data are expressed as the mean ± SEM, * *p* < 0.05, ** *p* < 0.01, *** *p* < 0.001, ns *p* > 0.05.

Together, CO poses an anti-dyslipidemia effect and regulates the metabolic phenotype by inhibiting the concentration of serum BCAA. The gene and protein expression of PPARγ related to lipid biosynthesis, the specific regulation of Stearoyl ethanolamide, Mesalamine, Lipoic acid, Picrotin, Citicoline, Norbuprenorphine, and Oxoamide make a big effort to alleviate dyslipidemia in HFD mice under the treatment of CO. We concluded that CO regulating of metabolic phenotype alleviates dyslipidemia in high fat-fed mice, in particular, Ile and Leu mediated the regulation of arachidonic acid and steroid biosynthesis metabolism (Figure 7).

## Figures and Tables

**Table 1:** Quantitative PCR primers used in this study.

Gene	Forward (5′-3′)	Reverse (5′-3′)
PPARγ	CCATTCTGGCCCACCAAC	AATGCGAGTGGTCTTCCATCA
β-actin	GTCCACCTTCCAGCAGATGT	GAAAGGGTGTAAAACGCAGC

**Table 2:** The relative content of Camellia (*Camellia oleifera Abel*.) seed.

Fatty Acid	Relative Content
Myristic Acid (C14:0)	0.09%
Palmitic Acid (C16:0)	0.09%
Palmitoleic Acid (C16:1n7)	81.30%
heptadecanoic acid (C17:0)	0.59%
10-Heptadecenoicacid (C17:1n7)	0.35%
Stearic Acid (C18:0)	6.27%
oleic acid (C18:1n9c)	0.06%
linoleic acid (C18:2n6c)	0.04%
Linolenic Acid (C18:3n3)	8.01%
Arachidic Acid (C20:0)	0.09%
11-Eicosenoic Acid (C20:1)	3.12%

## Data Availability

The data used to support the findings of this study are available from the corresponding author upon request.

## Supplementary Materials

The following supporting information can be downloaded at: https://www.mdpi.com/article/10.3390/nu14122424/s1, Table S1: The putative important 27 biomarkers from CO and HFD groups.
