# Supplementary material for: Camellia (Camellia oleifera Abel.) Seed Oil Regulating of Metabolic Phenotype and Alleviates Dyslipidemia in High Fat-Fed Mice through Serum Branch-Chain Amino Acids
Source: Nutrients. 2022 Jun 10;14(12):2424. doi: 10.3390/nu14122424 (PMC9228151; doi:10.3390/nu14122424)
Supplement: Supplementary file 1 [file nutrients-14-02424-s001.zip › nutrients-1735172-supplementary.pdf]

Supplementary Table S1. The putative important 27 biomarkers from CO and HFD groups

Sample	CO1	CO2	CO3	CO4	CO5	CO6	HFD1	HFD2	HFD3	HFD4	HFD5	HFD6	Up/down
**Label**	CO	CO	CO	CO	CO	CO	HFD	HFD	HFD	HFD	HFD	HFD	
**Stercobilin**	45010573.1	171751440	80862147	81524869.8	100672527	163521248	38798412.3	30665389.2	62832058.8	35797629.7	13458515.9	26705898.2	up
**Glycoursodeoxycholic acid**	230803202	46107324.1	76964606.7	142816552	77983471.2	103578139	865704.301	18280030.4	27931944.2	75257220.9	16006657.5	76016178.5	up
**Muricholic acid**	28619353.7	8514685.3	6331478.28	5042767.22	28793375.1	47425799.1	292139.697	1477651.88	10442825	8962120.72	328220.795	2910308.99	up
**Menaquinone**	9330015.64	7545703.71	7245053.8	4348502.52	16690058.65	8680282.71	986069.623	2298372.695	1677475.477	3838188.127	6705763.816	5546175.118	up
**Phosphoethanolamine**	28990257.4	31314321.7	19423817.9	33407580.6	19178784.71	58595058.4	35242061.2	4397689.261	11788624.26	13686239.39	2625400.111	4704473.927	up
**Stearoyl ethanolamide**	75628126.2	22329284.1	199432722	47900519.4	36662389.66	331897452	14599463.56	6120978.918	14126766.49	23679605.06	7417231.673	8196511.835	up
**Glycerophospho-N-palmitoyl**	23529344.1	33497855.3	142376686	107915720	6237766.34	114464099	6568269.09	2765073.768	1902798.592	68442088.59	9986249.518	33219894.57	up
**Noradrenaline**	3061989.92	3040592.02	1266780.94	2574557.98	3193775.04	2688354.39	10247599.4	6126485.69	4482196.32	4012592.6	2853377.82	4000016.01	down
**Prostaglandin H2**	7070530.06	6810632.44	5157759.92	5886891.6	5353288.73	5289075.57	33263156.2	34272123.4	14174798.6	9985791.11	6618105.24	11948356.1	down
**Mesalamine**	2259394.71	5018632.7	2891796.9	2484760.26	3070549.16	1772480.72	11657042.9	4977439.96	3723044.41	6301457.61	3726517.86	3941315.89	down
**Sepiapterin**	721836.297	1228372.58	285443.406	655482.132	544233.832	350445.39	1634543.75	1731311.64	905673.063	554372.312	4045100.4	543534.01	down
**Lipoic acid**	130200.588	346558.337	190768.954	184701.362	253719.704	129957.152	5883048.95	546317.763	2372279.9	401482.925	645213.482	90622.0975	down
**lipoamide**	1043798.68	1655638.35	957635.278	1227148.59	791469.968	1040463.21	5821412.86	2359531.92	2809645.31	1133581.21	4654483.83	572765.586	down
**Picrotin**	60414.4695	118244.028	40202.8904	68882.1001	72449.388	48783.0722	779016.287	96383.5073	7421524.76	115594.83	206592.228	71785.5189	down
**Isoferulic acid**	42566.3915	250740.1	1291605.25	43744.8705	69360.608	74416.7605	824622.71	238283.741	5074748.28	758328.064	1204863.66	150707.105	down
**Citicoline**	12680.7092	39897.46	15003.0419	15220.8631	17029.4093	11687.3945	61407.8967	30036.3982	711664.302	24293.8122	75188.7943	15105.6847	down
**isoleucine**	4136364.31	10274487.4	2038443.77	3525649.97	2299851.55	1204301.18	5981776.87	12478718.6	7682079.97	3814535.01	16345376.2	3462728.34	down
**7-Methyladenine**	267306549	617907986	104680047	76446679.4	354452125.5	209707921	480080547.3	36526411.58	33696631.44	25195950.66	15870806.25	37893575.46	down
**Testosterone**	8207577.88	8167624.14	4385277.7	8057264.13	3277972.111	4225735.09	14172821.68	15524136.07	9730150.265	16052029.58	13344369.57	15102070.1	down
**Hydroquinone**	24684407.8	25480001.1	21871716.3	22966776.9	27191407.19	25642573.5	68938256.06	69646102.29	34587928.72	30967805.98	42942360.62	40601481.21	down
**Dimethylglycine**	2552279.35	8217876.72	2037969	5946616.3	5756244.245	3830538.4	11091923.96	3841793.821	45009956.32	5005992.775	6320003.927	6674947.554	down
**Norbuprenorphine**	1106346.18	1819324.04	1476923.99	1516286.91	1565078.827	508391.275	3557951.445	3294346.332	436251.0637	2562871.259	3122821.951	3493034.758	down
**Oxoamide**	5972088.29	12379838.5	4699982.69	12876668.5	8127032.468	2757716.63	30895945.57	12276404.37	5151542.893	8586247.034	11887655.65	22533786.18	down
**Acetophenone**	3261267.99	3783561.28	2300946.45	2397512.81	3387909.796	2912523.52	9506173.173	48903953.34	8119470.436	3152933.773	5593720.805	3910723.761	down
